# Stop antibiotics when you feel better? Opportunities, challenges and research directions

**DOI:** 10.1093/jacamr/dlae147

**Published:** 2024-09-09

**Authors:** A J Borek, A Ledda, K B Pouwels, C C Butler, G Hayward, A S Walker, J V Robotham, S Tonkin-Crine

**Affiliations:** Nuffield Department of Primary Care Health Sciences, University of Oxford, Oxford, UK; National Institute for Health and Care Research (NIHR) Health Protection Research Unit in Healthcare Associated Infections and Antimicrobial Resistance, University of Oxford, Oxford, UK; National Institute for Health and Care Research (NIHR) Health Protection Research Unit in Healthcare Associated Infections and Antimicrobial Resistance, University of Oxford, Oxford, UK; Clinical and Public Health, UK Health Security Agency, London, UK; National Institute for Health and Care Research (NIHR) Health Protection Research Unit in Healthcare Associated Infections and Antimicrobial Resistance, University of Oxford, Oxford, UK; Nuffield Department of Population Health, University of Oxford, Oxford, UK; Nuffield Department of Primary Care Health Sciences, University of Oxford, Oxford, UK; National Institute for Health and Care Research (NIHR) Health Protection Research Unit in Healthcare Associated Infections and Antimicrobial Resistance, University of Oxford, Oxford, UK; Nuffield Department of Primary Care Health Sciences, University of Oxford, Oxford, UK; National Institute for Health and Care Research (NIHR) Health Protection Research Unit in Healthcare Associated Infections and Antimicrobial Resistance, University of Oxford, Oxford, UK; Nuffield Department of Medicine, University of Oxford, Oxford, UK; NIHR Biomedical Research Centre, Oxford, UK; National Institute for Health and Care Research (NIHR) Health Protection Research Unit in Healthcare Associated Infections and Antimicrobial Resistance, University of Oxford, Oxford, UK; Clinical and Public Health, UK Health Security Agency, London, UK; Nuffield Department of Primary Care Health Sciences, University of Oxford, Oxford, UK; National Institute for Health and Care Research (NIHR) Health Protection Research Unit in Healthcare Associated Infections and Antimicrobial Resistance, University of Oxford, Oxford, UK

## Abstract

Shortening standard antibiotic courses and stopping antibiotics when patients feel better are two ways to reduce exposure to antibiotics in the community, and decrease the risks of antimicrobial resistance and antibiotic side effects. While evidence shows that shorter antibiotic treatments are non-inferior to longer ones for infections that benefit from antibiotics, shorter courses still represent average treatment durations that might be suboptimal for some. In contrast, stopping antibiotics based on improvement or resolution of symptoms might help personalize antibiotic treatment to individual patients and help reduce unnecessary exposure. Yet, many challenges need addressing before we can consider this approach evidence-based and implement it in practice. In this viewpoint article, we set out the main evidence gaps and avenues for future research.

## Opportunities of stopping antibiotics when you feel better

Exposure to antibiotics accelerates antimicrobial resistance (AMR) and impacts the human microbiome.^1–3^ AMR is considered to spread through collateral selection of resistant genes rather than stopping antibiotics ‘too soon’ (Box 1).^4,5^ Antibiotic use increases risks of subsequent infections being resistant to antibiotics,^6^ which, in turn, makes treatment failure more likely and recovery slower, compared to non-resistant infections, and increases workload in primary care.^7,8^ Thus, reducing unnecessary antibiotic exposure is paramount for individuals, populations and healthcare services. It could help prevent AMR and reduce antibiotic side effects, which is important to patients.^9^

Box 1. **Common beliefs on antibiotic use and the development of AMR.**

There is a widespread belief that resistance develops because untreated resistant pathogens re-emerge when antibiotics are stopped ‘too soon’ before pathogens are ‘wiped out’. This is because pathogens exposed to the drug, but not killed by it, could develop ways to ‘resist’ the drug. For a long time, this belief supported the clinical practice of prescribing longer antibiotic courses and the advice to ‘always finish antibiotic courses’. If a patient were a Petri dish with only the bug causing the infection, then the best course of action would be to take antibiotics until there is no pathogen left. However, the patient is a complex ecosystem with a lot of different microbes and with an immune system. Antibiotic use exposes commensals and increases their chances of developing resistance through so-called ‘collateral selection’. Thus, an antibiotic course needs to be long enough to treat the infection, without relapse, while minimizing the risks to the rest of the microbiome. This approach supports the recent calls to shift clinical practice from longer to shorter antibiotic courses and the advice from ‘always complete the course’ to ‘always take as advised’ by the clinician.^1–4^

The preferred approach involves *not prescribing antibiotics* when they are likely to be of no or limited benefit—which is for most infections presenting in primary care. When antibiotics are needed [e.g. for some urinary tract infections (UTIs) and pneumonia], *shorter antibiotic courses* help minimize unnecessary exposure.^10^ For most infections, shorter treatment duration is non-inferior to longer courses,^10–14^ and—at least in some instances—is associated with a lower abundance of resistant genes.^12,15^ However, recommended durations of antibiotic treatments are not always evidence-based^13^ and are often exceeded by prescribers.^16^ They also represent *average* treatment courses to support recovery for *most* patients; they are not necessarily optimal for everyone because individual patients and infectious episodes respond differently to different treatment durations.^11^ This might lead to under- or over-treating some patients.^10^

A better alternative could be to *personalize antibiotic treatment*, using the minimum duration that leads to the individual patient’s recovery. This might involve stopping antibiotics when patients ‘feel better’, i.e. based on the improvement or resolution of their symptoms.^4,5,17^

Stopping antibiotics when better is not as revolutionary as it may initially sound. Variations in how clinicians prescribe antibiotics^18^ and patient non-adherence are common.^19,20^ Some patients already stop antibiotics when they feel better, miss doses and seek to limit antibiotic use without clinical supervision.^21^ Some primary care clinicians and patients in England already use the ‘stop when better’ approach and others are open to the idea for UTIs.^9^ Primary care professionals in Catalonia also supported this idea for respiratory infections, with AMR experts most amenable to it.^22^ Moreover, as antibiotics continue to be prescribed for various other (often social) reasons for viral infections, stopping antibiotics when better could reduce exposure without risks of treatment failure.

## Challenges and research directions

Nevertheless, some concerns and uncertainties about this approach require further exploration. ‘Stopping antibiotics when better’ contradicts widespread and almost ingrained beliefs that ‘completing’ antibiotic courses is necessary to avoid AMR. While the World Health Organization has moved away from the ‘finish your prescription’ slogan, the change in public health messaging and people’s beliefs lags.^23^ It is still in the UK school curriculum!^24^ Patients and prescribers want to be reassured that this new approach is supported by evidence on efficacy and, critically important, does not put patients at risk of harm.^9^ Such evidence is still lacking specifically for ‘stopping when better’. The key challenge involves identifying when exactly antibiotics can be safely stopped based on symptoms to minimize chances of relapse. We currently lack evidence on how pathogen burden is associated with symptoms experienced by individuals: how patients feel when pathogen levels are low enough to minimize relapse.

Another challenge involves operationalizing ‘feeling better’ to give clear and specific advice that is what clinicians and patients want.^9,22^ ‘Feeling better’ is likely to vary between people, infections and symptoms. It may mean an improvement in (certain) symptoms or full recovery from (certain/all) symptoms. For example, fever might be a more relevant symptom to guide stopping antibiotics than cough that may take longer to resolve but without impacting on daily activities. Some patients with UTIs preferred to stop antibiotics after symptoms had fully resolved rather than when they improved—they considered ‘symptom improvement’ ambiguous and the threshold for stopping treatment unclear.^9^ Differences in experiencing symptoms and their relative importance were also apparent (e.g. pain versus need to urinate).^9^ A clear and feasible definition of when patients could/should stop antibiotics is needed to help clinicians give appropriate and unambiguous advice. This should be used as part of shared decision-making and include communication strategies effective when not prescribing antibiotics, such as discussing concerns and expectations, natural history and progression of acute infections, and safety-netting advice.

There is also a need to identify the clinical contexts for which this approach would be suitable and beneficial, which may vary between syndrome, causative agent, patient characteristics as well as antibiotic choice. However, this would require rapid point-of-care tests in primary care to identify pathogens, which is complicated by many factors, including the presence of commensals.

Despite the potential benefits of personalizing antibiotic treatment based on symptoms, no studies have yet established the efficacy and safety of this approach in primary care. Research is needed to address this lack of evidence and other challenges to personalizing antibiotic treatment. While qualitative,^9,22^ modelling and experimental^25^ studies start addressing these gaps, we highlight the main research directions (Box 2), complementing the viewpoint by Llor *et al*.^17^ on this topic. While investigating different treatment durations, studies should include detailed, frequent patient symptom diaries of how/when symptoms change to identify the range of individual responses to treatment and how they differ between patients (e.g. based on age, sex and comorbidities), infection types and pathogens. They should also include frequent laboratory tests to determine the pathogen abundance at different points, during and after treatment, and estimate associations with symptoms. Patients should be followed for longer (>1 year, potentially through data linkage) to record infection recurrences and any antibiotic sensitivities of those infections. This matching of the symptoms, pathogen levels and response to treatment (recovery) could help define an optimal point for stopping antibiotics based on symptoms. Finally, improved rapid tests could help determine pathogen identification and antimicrobial sensitivity in a way that is actionable at the point of care, and further inform treatment approaches.

Box 2. **Main evidence gaps and research questions on personalized, symptom-based antibiotic treatment durations in primary care.**

For different infections, particularly those that usually require antibiotics (e.g. UTIs and pneumonia)*

Individual responses to antibiotic treatments

- How is pathogen abundance associated with symptoms?
- How/when do symptoms change during antibiotic treatment?
- How do individual responses to antibiotic treatment differ between patients, infections and pathogens?
- What are the optimal treatment durations for different infections and patients (e.g. based on risk factors)?
- How do antibiotic treatments, with different durations, affect human microbiome?

Efficacy and safety of stopping antibiotics when feeling better

- Is this approach efficacious, effective and safe in terms of antibiotic use, clinical outcomes and AMR?
- How does it compare to using standard courses (recognizing that some patients do not complete standard courses)?
- For which patients and infections is it safe or not safe?
- For which pathogens is this approach more/less appropriate and effective?
- How common are adverse effects (e.g. deterioration, relapse and recurrence) and for whom?

Operationalizing symptom-based treatment durations and advice

- Which symptoms should help determine when it is safe to stop treatment?
- When is it appropriate to stop antibiotics safely—when symptoms improve (and how much) or fully resolve?
- How should prescribers communicate the advice to stop antibiotics based on symptoms to patients?
- How should prescribers communicate about personalized, symptom-based antibiotic treatment, as part of shared decision-making and alongside other communication strategies relevant to discussing infections and antibiotics in primary care?

Awareness of symptom-based antibiotic treatment approaches

- How can we raise public awareness of how AMR develops and how reducing antibiotic exposure helps prevent resistance rather than completing antibiotic courses (a strategy for which there is little/no evidence)?

Implementation of symptom-based antibiotic treatment approaches

- How should antibiotics be packaged to support personalized treatment approaches?
- How should we ensure safe disposal of leftover antibiotics?

*Stopping antibiotics when better could also be trialled for infections that do not usually require antibiotics but when antibiotics are often over-prescribed, such as respiratory tract infections, due to clinical uncertainty or for other reasons (e.g. risk avoidance, patient demand/pressure and patient satisfaction).

Such key uncertainties around stopping antibiotics based on symptoms need to be addressed before this approach can be considered evidence-based and implemented. If it were to be widely used, implications for clinician and patient behaviours (e.g. advice giving and disposal of leftover antibiotics) and for antibiotic packaging would also need addressing. Nevertheless, personalized approaches to antibiotic treatment based on perhaps the most sensitive assessment of recovery we have, namely, how patients themselves feel, offer a potentially transformative way to balance effective treatment with minimizing exposure and helping prevent AMR.
